# *In vivo* antiplasmodial activities of stem bark extracts of *Avicennia marina* in *Plasmodium berghei*-infected mice

**DOI:** 10.11604/pamj.2023.44.93.38448

**Published:** 2023-02-16

**Authors:** Gitau Wilfred, Edinah Kwamboka Songoro, Jeremiah Waweru Gathirwa, Francis Kimani, Humphrey Njaanake Kariuki

**Affiliations:** 1 Department of Medical Microbiology, Jomo Kenyatta University of Agriculture and Technology, Nairobi, Kenya,; 2 Centre for Traditional Medicine, Drugs and Research, Kenya Medical Research Institute, Nairobi, Kenya,; 3 Centre for Biotechnology, Research Development, Kenya Medical Research Institute, Nairobi, Kenya,; 4 Department of Medical Microbiology, University of Nairobi, Nairobi, Kenya

**Keywords:** Malaria, *Avicenia marina*, *P. berghei*, mice, suppression

## Abstract

**Introduction:**

malaria remains the leading cause of morbidity and mortality in developing tropical and subtropical nations. Due to the emergence and spread of drug resistance to currently available drugs, there is a need for the search of novel, safe, and reasonably affordable anti-malarial medications. The objective of this study was to assess the in vivoanti-malarial effectiveness of Avicennia marina stem bark extracts in a mice model.

**Methods:**

guidelines 425 of the Organization for Economic Cooperation and Development were used to determine the extracts' acute toxicity. Mice infected with chloroquine-sensitive Plasmodium berghei (ANKA strain) were tested for in vivoanti-plasmodial activity, and by giving oral doses of 100 mg/kg, 250 mg/kg, and 500 mg/kg body weight of extracts, the plant's suppressive, curative, and preventive effects were assessed.

**Results:**

mice treated with dosages of up to 5000 mg/kg showed no evidence of acute toxicity or mortality. Consequently, it was determined that the acute lethal dosage of Avicennia marina extracts in swiss albino mice was greater than 5000 mg/kg. All doses of the extracts exhibited significant (p<0.05) dose-dependent suppression of P. berghei in the suppressive tests compared to the control group. At the highest dose (500 mg/kg), Methanolic crude extracts exerted the highest (93%) parasitemia suppression during the 4-day suppressive test. The extracts also displayed significant (p<0.001) prophylactic and curative activities at all doses compared to the control.

**Conclusion:**

results from this study ascertained the safety and promising curative, prophylactic and suppressive anti-plasmodial capabilities of the stem bark extracts of Avicennia marina in mice model.

## Introduction

Malaria is an infectious, life-threatening disease caused by *Plasmodium* (*Plasmodium vivax, Plasmodium malariae, Plasmodium ovale*, and *Plasmodium knowlesi*) species which are transmitted through the bites of infected female *Anopheles* mosquitoes [1]. The disease remains a major source of morbidity and mortality, especially in tropical developing countries like Kenya, where children under 5 and pregnant women are most at risk [2]. Globally, nearly half the population is at risk of contracting malaria, with African regions bearing the highest burden [3]. According to World Health Organization´s (WHO´s) 2019 report, an estimated 228 million cases of malaria infections were reported globally with 93% of those cases taking place in African region, followed by the WHO South-East Asia Region (3.4%) and the WHO Eastern Mediterranean Region (2.1%) [4]. Of the estimated 405,000 malaria-related deaths globally, 67% were children under the age of five, with 94% of the fatalities occuring in the WHO African Region [4]. With an estimated 3.5 million new clinical cases and 10,700 deaths each year, malaria is still a major public health concern in Kenya, accounting for 13% to 15% of outpatient visits [5].

Since the isolation of quinine in 1820s as the first effective anti-malarial drug, a number of other natural and synthetic compounds have been developed for treatment of the disease [3]. For example, chloroquine, a synthetic compound of quinoline was introduced in the 1940s to treat all forms of malaria and the population-based prophylaxis with this drug was initiated in many areas in the 1960s [3]. Despite widespread resistance to the drug throughout the tropics in the 1980s, chloroquine treatment for uncomplicated *P. falciparum* malaria is still utilized in some countries where the strains are sensitive [6]. Currently, WHO Model List of Essential Medicines has listed 14 drugs for curative treatment, and 4 for prophylaxis against malaria, formulated either as single compounds or as combinations [7]. WHO has recommended artemisinin, in combination with drugs such as amodiaquine, lumefantrine, mefloquine, and sulphadoxine-pyrimethamine as first-line treatment of uncomplicated falciparum malaria and parenteral artesunate for the treatment of severe malaria in endemic zones [8]. In sub-Saharan African, pyrethroid insecticides are used in malaria prevention efforts, both for indoor residual spraying (IRS) and long-lasting insecticide nets (LLINs) [9]. Other strategies used to lower the number of malaria cases, include entomologic surveillance, and intermittent preventative therapy for expectant mothers in malaria-endemic areas [10]. However, the emergence of pyrethroid-resistant mosquitoes in Western Kenya, prompted the government to stop spraying insecticide against mosquitoes between 2013 and 2016 [11]. Malaria parasites resistant to quinine and antifolate derivatives has been widely reported [12]. The emergence and geographic expansion of artemisinin resistant *P. falciparum* in the Greater Mekong Subregion (GMS) poses a severe challenge in efforts to control and eradicate malaria globally [13]. Studies conducted recently have established the rise of partial resistance to artemisinin in some African regions, notably in Eritrea, Rwanda, and Uganda [14,15]. Given Africa's extensive reliance on artemisinin-based combination therapies (ACTs) as the first-line treatment of uncomplicated malaria, the issue of artemisinin partial resistance and partner drug resistance must be closely monitored and addressed urgently through innovative development of novel anti-malarial drugs [8,16].

Globally, plants have extensively been used in traditional medicine to treat illnesses including malaria, fever, cholera, diarrhoea, pain, breast swelling, elephantiasis, inflammation, flu, dyslipidemia and hyperglycaemia particularly in endemic areas [17-21]. Artemisinin and quinine, the two main groups of the most effective antimalarial drugs, are derived from plants *Artemisia annua* and *Cinchona sp*., respectively [22]. In an effort to find new natural antimalarial medicine in Kenya, several studies have been conducted on various plants which are traditionally used by the local communities to treat malaria [11,23,24].

The seashore ecosystem serves as a source of food, medicine, fuel, and building materials to local communities and one of the widely distributed plants along seashores is mangrove [21,25]. Mangroves have extensively been explored for their versatile medicinal uses which include the treatment of abscesses, ulcers, flu, rheumatism, smallpox, cancer, malaria, scabies, sores and boils [25-31].

*Avicennia marina* (Forsk.) Vierh, (gray mangrove) is one of the mangrove species which is widely distributed within a latitudinal range of 30°N to 38°S with a global distribution in intertidal sections of seabeds, estuaries, rivers, and streams [32,33]. Several studies evaluating the antiplasmodial activities of *A. marina* tree extracts have been conducted with results showing varying degrees of efficacy [8,34]. Previous research has also revealed the presence of phytochemicals such as saponin, alkaloids, and phenolic contents, all of which have antimalarial properties [26,29].

Despite its widespread presence on the Kenyan coast, our investigations revealed that no documented *in-vivo* research into the antiplasmodial efficacy of African *A. marina* has been conducted, resulting in a knowledge gap due to a lack of evidence-based data. As a result, the current study sought to identify phytochemicals as well as assess the safety and antiplasmodial efficacy of Kenyan *Avicennia marina* in a mice model.

## Methods

**Plant materials:** stem barks of *Avicennia marina* were collected from Gazi bay mangrove ecosystem in Kwale County, coastal Kenya. The Department of Botany Herbarium at the University of Nairobi's, School of Biological Sciences performed the identification and authentication of the plant. For documentation, a voucher specimen was placed in the herbarium (voucher number JNM 2019/02). The barks were air dried for five days under shade.

**Preparation of extracts:** the extraction was done using procedure described by Okaiyeto *et al*. [35]. Briefly, the dried stem barks were ground to powder using an electric miller. One hundred (100) gm of the powder was soaked in 500 mL of methanol for 48 hours at room temperature with frequent stirring in a 1 L volumetric flask. Whatman filter paper no. 1 was used to filter the mixture, and the filtrate was collected in a stoppered conical flask. For hot decoction, 100 g of the plant material was soaked in 500ml of distilled water and the mixture was boiled for 20 minutes. The mixture was filtered with Whatman filter paper no. 1 into a stoppered conical flask after being allowed to cool. Cold maceration was used to prepare the cold aqueous extract where 100 g of the powder was mixed with 500 ml of distilled water at room temperature. The mixture was extracted for 48 hours with continuous agitation using a magnetic stirrer. It was then filtered through Whatman filter paper no.1 and the filtrate collected in a 1 liter stoppered conical flask.

**Concentration of the extracts:** in order to get solid residue, the filtrates from organic solvents were concentrated in a rotary vacuum evaporator (buchi rotavapor R-205), and then freeze-dried (-20°C). Freeze-drying was used to concentrate the filtered aqueous extracts. Using the following formula, the extraction percentage yield was calculated [36]:


$$Percentage\text{ of extraction = }\frac{W2-W1(g)}{W0(g)}*100$$

Where W_1_= the weight of the empty container; W_2_= the weight of the container plus the weight of the plant extract; W_0_= the weight of the milled extract (100gm).

**Phytochemical screening:** a qualitative phytochemical analysis to identify important bioactive molecules such as steroids, coumarins, alkaloids, saponin, tannin, flavonoid, terpenoids, and anthraquinones was carried out using standard methodologies [37].

**Experimental animals:** a total of ninety (90) Swiss albino female mice weighing 18 to 22 grams and aged 5 to 6 weeks were procured from The Kenya Medical Research Institute´s (KEMRI's) animal house in order to conduct toxicity and antimalarial assays. They were housed in standard Macrolon type II cages for 12 hours at a constant room temperature of 25 to 27 degrees Celsius and a relative humidity of 60 to 70 percent, with unlimited access to food and water. Animals were moved from the unit to the laboratory at least 30 to 60 minutes before use in order to reduce the impact of stress on them. Permission and approval for animal use were obtained from the Kenya Medical Research Insitute Animals Care and Use Committee.

**Experimental parasites and inoculation procedures:** cryopreserved *P. berghei*, were obtained from KEMRI´s Center for Traditional Medicine Research (CTMDR) laboratory. Serial passage of *P. berghei* was initiated by administering 2 × 10^7^ (0.2ml) of blood-containing parasites intraperitoneally (i.p.) into albino mice and allowing the rodents to develop the parasites for 7 days. While under anaesthesia induced by halothane in a closed chamber, 1 to 2 ml of blood was drawn via cardiac puncture into a heparinized vacutainer tube. Based on the parasitaemia level of the donor mice and the red blood cell (RBC) count of normal mice, blood was diluted with sterile, non-pyrogenic 0.85% physiological saline so that 1 ml of blood contains 5 × 10^7^ infected red blood cell (RBC). Zero point two ml of this diluted blood, which contained 1 × 10^7^ *P. berghei*-infected RBCs, was administered intraperitoneally into each test- and control-group mice [38].

***In vivo* acute toxicity test:** in accordance with Organization for Economic Cooperation and Development (OECD) Guideline no. 425 [39], non-infected female Swiss albino mice were used to test the acute toxicity of crude methanol and acqueous extracts of *Avicennia marina* on the animals. The mice were 5-6 weeks old and weighed 18-22 g. Five mice were fasted overnight but were allowed water *ad libitum* and were weighed before testing. Briefly, a single dose of 5000 mg/kg (0.2 ml) of each extract (aqueous and methanol) was given orally to a female mouse. Food was restricted for a further two hours after extract administration. The mouse was observed continuously for 30 minutes for 4 hrs and thereafter 24 hrs. Since no deaths were noted within the first 24 hours, the additional four mice were given the same dosage of the extract (5000 mg/kg) and monitored for any signs of toxicity over the course of the following 14 days, such as decreased respiration, writhing, reduced motor activity, reduced body tone, and death.

**Pharmacological screening (Peter´s four-day suppressive test):** the schizontocidal activity of the crude extract against chloroquine-sensitive *P. berghei*-infected mice was assessed using Peter's 4-day suppression test [40]. Briefly, female Swiss albino mice weighing 18-22 g were intraperitoneally injected with 0.2 ml of infected blood on the first day (day 0). The mice were then randomly divided into six groups of five mice each. Groups I, II, III and IV were designated as test groups while groups V and VI were designated negative and positive control groups, respectively. The test groups received 0.2 mls of 100 mg/kg, 250 mg/kg, and 500 mg/kg crude extract of *A. marina* via oral gavage three hours after parasites inoculation, while the positive and negative control groups received chloroquine at a dose of 10 mg/kg and an equivalent amount of vehicle (0.2 ml distilled water) respectively for four consecutive days (day 0-3). A drop of blood was taken from each mouse's tail on the fifth day (day 4, 24 hours after the last dose, or 96 hours after infection), and it was used to make thin blood smears that were stained with 10% Giemsa. And by counting the number of parasitized erythrocytes in five randomly selected fields of a microscope using an oil immersion objective (100x magnification), the following equation was used to determine the parasitemia level [41]:


$$\%Parasitemia=\frac{Number\text{ of parasitized RBC}}{Total\text{ number of RBC counted}}*100$$

Average chemo-suppression by percentage was determined as [42]:


$$\text{Avarage chemo - suppression by percentage=100[}\frac{\text{A-B}}{\text{A}}\text{]}$$

Where A represents the average parasitaemia in the test group and B represents the average parasitaemia in the negative control group, respectively.

**Rane´s (curative) test:** according to Nardos *et al*. procedures [43], the crude extract's chemotherapeutic effectiveness against an already-established illness was evaluated. Briefly, mice were intraperitoneally injected with 0.2 ml of a standard inoculum containing 1 × 10^7^ *P. berghei*-infected erythrocytes on day 0 of the experiment. At day 3, mice were randomly assigned to two groups of positive and negative control (V and VI), and four test groups (I, II, III, and IV), each group with five mice. Positive and negative controls were given oral doses of 0.2 ml of 10 mg/kg of chloroquine and vehicle (distilled water), respectively. Each mouse's tail was processed into a thin blood film, air dried, and Giemsa stained on days 3 and 7 to track the parasitaemia level.

**Prophylactic (repository infection) test:** the procedure described by Peters [44] was modified slightly to examine the crude extracts' prophylactic activities against residual infection. Six groups of five mice each were randomly selected from the mouse population. Animals in group I (negative control) and II (positive control) were given 0.2 mls of vehicle (distilled water) and 0.2 ml (10 mg/kg) of chloroquine respectively. Exactly 0.2 ml of 100 mg/kg, 250 mg/kg, or 500 mg/kg of the extract were given orally to groups II through V. The mice in each group received treatment for 3 days in a row (D0 - D2), then on day 4 (D3), the 0.2ml infected blood was intraperitoneally injected into the mice. Within 72 hours of inoculation, thin films were made from the tail blood of each mouse, Giemsa stained to assess the parasite density.

**Monitoring mean survival time:** in all of the *in vivo* antiplasmodial experiments, mortality was tracked and the number of days from the moment of parasite inoculation up until death was recorded for each mouse in the treatment and control groups for 30 days. Each group's mean survival time (MST) was calculated as follows [45]:


$$MST=\frac{Sum\text{ of survival time of all mice in a group (in days)}}{Total\text{ number of mice in the group}}$$

**Monitoring body weight:** each mouse's body weight was recorded on days 0 (D0) and 4 (D4) of the 4-day suppressive test, one hour prior to infection on D0 and following treatment on D4, respectively. For Rane´s and repository test the body weight for each mouse was taken 3 hours before infection on day-0 (D0) and after treatment on day-7 (D7) to determine the efficacy of methanolic and aqueous crude extracts of *A. marina stem*bark on *P. berghei* infected mice [18]:


$$Percent\text{ body weight change = }\frac{Body\text{ weight day 4 - Body weight day 0}}{Body\text{ weight day 4}}*100$$

**Data quality control:** the experimental animals were randomly selected and marked with a permanent marker pen to permit individual identification. During preparation of blood smears, unique codes were assigned in frosted microscopic slide. Parasites count was done by 4 blinded experienced laboratory technicians.

**Data analysis:** data were analyzed using SPSS version 21.0 software and results of the study were expressed as a mean ± standard error of the mean (M ± SEM). One-way analysis of variance (ANOVA) was used to compare between multiple treatment groups followed by Scheffe´s and Tamhane´s T2 *post hoc* test. P-values less than 0.05 were regarded as significant.

**Ethical clearance:** approval to use animal models was sort from Animal Care and Use Committee (KEMRI-ACUC). Ethical review and approval was obtained from the Scientific Ethics Review Unit-Kenya Medical Research Institute (KEMRI/RES/7/3/1).

## Results

**Extract yields:** stem bark extracts yielded a total of 23.1 g (23.1%) and 21.82 g (21.82%) of dried aqueous crude extract and dried methanolic crude extract respectively.

**Phytochemical analysis:** *Aviccenia marina's* stem bark extracts were subjected to a qualitative phytochemical screening test, which revealed that the plant contains alkaloids, anthraquinones, anthrocyanides, tannins, saponins, triterpenoids, flavanoids, and cardiac glycosides but no phenols or steroids (Table 1).

**Table 1:** phytochemicals of *A. marina* extracts

Phytochemicals	Methanolic extracts	Aqueous extracts
Alkaloids	-	+
Phlobatanin	-	-
Anthrocyanides	+	-
Anthraquinines	+	+
Tannins	+	+
Saponins	-	+
Steroids	-	-
Triterpenoids	+	-
Glycosides	+	-
Flavonoids	-	+
Phenol	-	-

+: present; -: absent of metabolites

**Acute cytotoxicity:** in the *in vivo* acute toxicity test no mortality was observed between day 0 and day 24 h and day 14 following oral treatment with either aqueous or methanolic extracts of *A. marina* stem bark at a dose of 5000 milligrams per kilogram of body weight. Additionally, no overt poisoning symptoms such writhing, reduced motor activity, decreased body tone, decreased feeding behaviors, or decreased limb tone were noticed. Lethal dose fifty (LD_50_) was therefore assumed to be above 5000 mg/kg.

**Figure 1:**
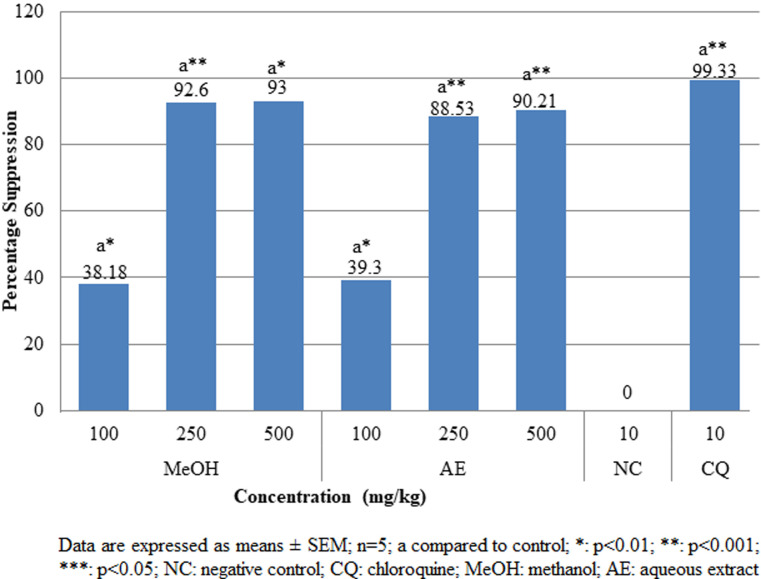
effects of *A. marina* crude extracts on parasitemia suppression of *P. berghei* infected mice in the 4-day suppressive test

**Figure 2:**
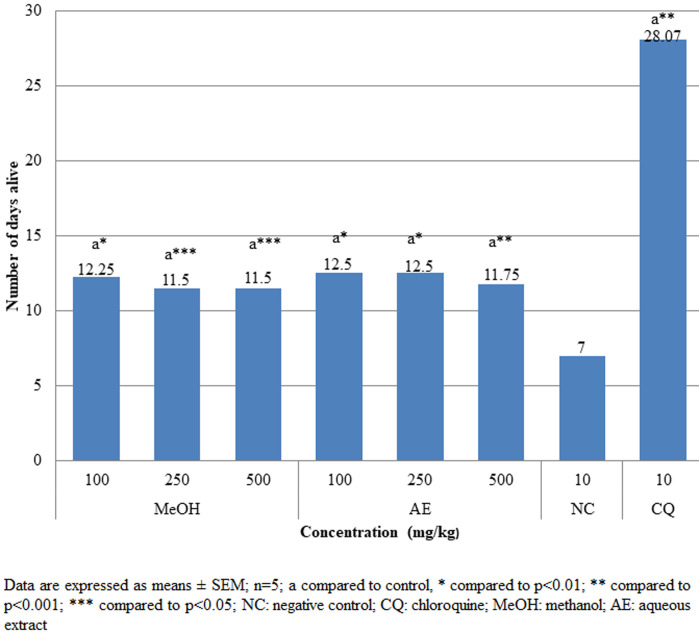
effects of *A. marina* crude extracts on mean survival time of *P. berghei* infected mice in the 4-day suppressive test

**Pharmacologic screening (Peter´s 4-day suppressive) test:** the findings of the 4-day suppressive test revealed that both aqueous and methanolic extracts had exceptional antiplasmodial action against female Swiss albino mice infected with chloroquine susceptible *P. berghei* as shown in Figure 1. Dose-dependent antischizoidal ability was demonstrated by all doses. The degree of suppression by aqueous extract after the 4-day test at dosages of 100, 250, and 500 mg/kg was 39.2%, 88.53%, and 90.20%, respectively, and that of methanol extract was 38.18%, 92.60% and 93%, respectively. Both aqueous and methanol extracts significantly (p<0.05) reduced parasitemia compared to negative control. At higher doses of 500 mg/kg, both methanolic and aqueous extracts showed significantly (p<0.001) higher percentage suppression of 93% and 90.21%, respectively compared to that of chloroquine (CQ) (10 mg/kg) (99.33%). The survival duration of the infected mice was significantly (p<0.05) extended by both the aqueous and methanolic stem bark extracts as compared to the negative control group (Figure 2).

**Effect of suppressive test on body weight of infected mice:** as shown in Table 2, none of the extract doses significantly (p>0.05) prevented mice from body weight loss in comparison to the negative control animals. However, the conventional medication (chloroquine 10 mg/kg) significantly (p<0.001) prevented body weight loss related to malaria compared to control mice.

**Table 2:** body weight change of infected mice treated with *Avicennia marina* extract in the suppressive test

Extract	Dose mg/kg body weight	D0 weight (g)	D4 weight (g)	Weight Change (%)
MeOH	100	21.25 ± 0.63	20.00 - 0.41	-6.26 ± 2.41
	250	20.50 ± 0.65	18.25 - 0.48	-12.38 ± 2.73
	500	21.50 ± 0.65	19.25 - 0.63	-12.08 ± 5.40
AE	100	21.00 ± 0.41	19.00 ± 0.41	-2.28 ± 6.43
	250	21.50 ± 0.29	20.25 ± 0.75	-6.55 ± 3.56
	500	20.50 ± 0.29	19.25 ± 0.48	-6.60 ± 1.51
NC	10	21.13 ± 0.15	18.40 ± 0.41	-15.71 ± 2.41
CQ	10	21.07 ± 0.27	23.00 ± 0.37	8.13 ± 1.68^a**^

Data are expressed as means ± SEM; n=5; ^a^: compared to control, *: p<0.01; **: p<0.001, ***: p<0.05; D0 to pre-treatment value on day zero, D4 to post-treatment value on day four; NC: negative control; CQ: chloroquine; MeOH: methanol; AE: aqueous extract

**Figure 3:**
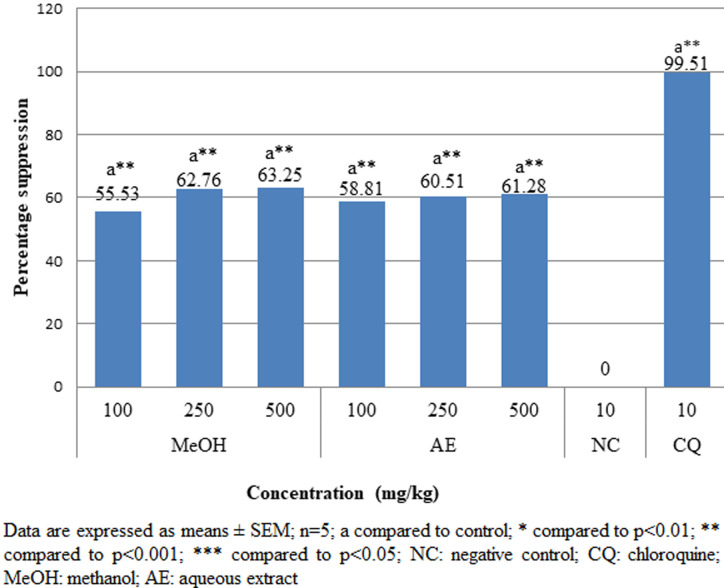
effects of *A. marina* crude extracts on parasitemia suppression of *P. berghei* infected mice in the curative test

**Figure 4:**
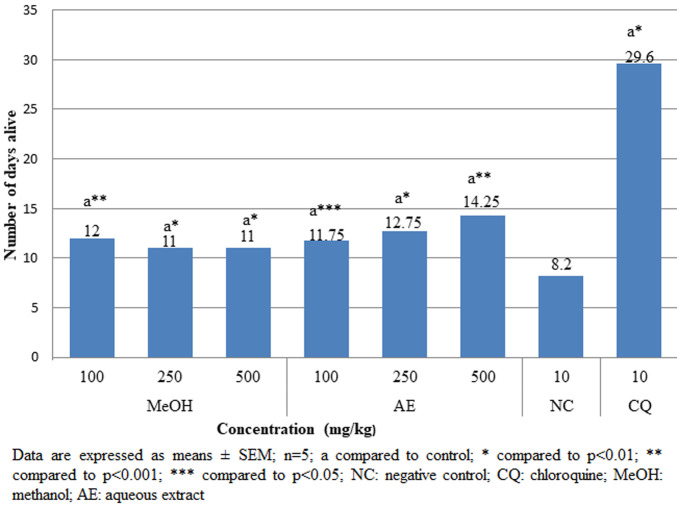
effects of *A. marina* crude extracts on mean survival time of *P. berghei* infected mice in the curative test

**Rane´s (curative) test:** when compared to the group that received a vehicle treatment, both aqueous and methanolic crude extracts significantly (p<0.001) reduced parasitemia in a dose-dependent manner (Figure 3). In comparison to the negative control, survival time was significantly (p<0.05) prolonged in all doses of the test sample (Figure 4). However, compared to CQ 10mg/kg, the extracts' therapeutic impact was less potent.

**Effects of curative test on body weight of infected mice:** in comparison to the negative control, all doses significantly (p<0.05) prevented the mice from losing body weight (Table 3). The body weight gain, however, was not dose-dependent.

**Table 3:** body weight change of infected mice treated with *Avicennia marina* extract in the curative test

Extracts	Dose (mg/kg)	D0 weight (g)	D7 weight (g)	Weight change (%)
MeOH	100	20.25 ± 0.48	17 ± 0.58	-19.27 ± 1.97^a***^
	250	19.75 ± 0.85	17.25 ± 0.63	-14.52 ± 2.94^a*^
	500	21.50 ± 0.28	18 ± 0.41	-18.17 ± 1.80^a*^
AE	100	21.00 ± 0.41	18.00 ± 0.41	-16.78 ± 2.56^a*^
	250	20.25 ± 1.03	17.00 ± 1.15	-19.56 ± 2.57^a***^
	500	20.75 ± 0.49	18.50 ± 0.29	-12.21 ± 2.62^a**^
NC	10	20.60 ± 0.51	15.40 ± 0.60	-36.67 ± 4.58
CQ	10	20.60 ± 0.51	20.60 ± 0.81	0.81 ± 1.99^a**^

Data are expressed as means ± SEM; n=5; ^a^ compared to control; *: p<0.01; **: p<0.001; ***: p<0.05; D0 to pre-treatment value on day zero; D7 to post-treatment value on day seven; NC: negative control; CQ: chloroquine; MeOH: methanol; AE: aqueous extract

**Figure 5:**
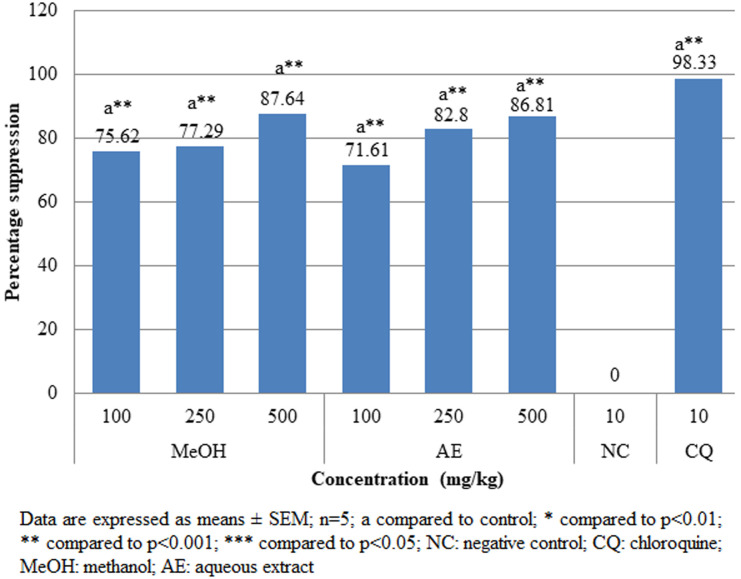
effects of *A. marina* crude extracts on parasitemia suppression of *P. berghei* infected mice in the prophylactic test

**Figure 6:**
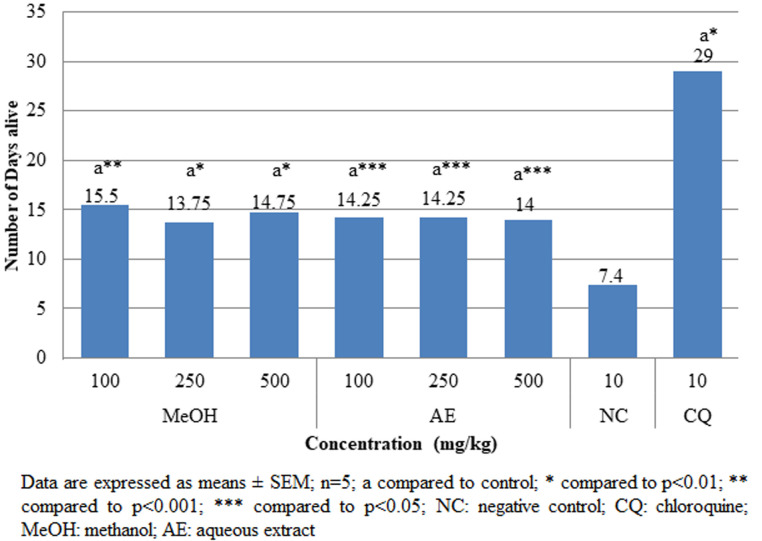
effects of *A. marina* crude extracts on mean survival time of *P. berghei* infected mice in the prophylactic test

**The prophylactic (repository) test:** when compared to the negative control, all doses of aqueous and methanolic crude extracts significantly (p<0.001) reduced parasite burden (Figure 5). Although total parasite suppression was not achieved, methanol (MeOH) 500mg/kg showed the highest levels of parasitemia suppression (87.64%) among the experiments. At all doses of the aqueous and methanolic extracts of the stem barks of *A. marina*, there was a significant (p<0.05) difference in the mean survival time between test mice and the negative control group (Figure 6).

**Effects of prophylactic test on body weight of infected mice:** when compared to the vehicle in all doses, methanolic crude extracts significantly (p<0.001) reduced weight loss in infected mice. When compared to the vehicle group, weight loss in test mice was significantly (p<0.01) reduced by both the aqueous extract (AE) 250mg/kg and AE 500mg/kg extracts. Aqueous extract (AE) 100mg/kg extract, however, was unable to prevent the test mice from losing weight (Table 4).

**Table 4:** body weight change of infected mice treated with *Avicennia marina* extract in the prophylactic test

Extract	Dose (mg/kg)	D0 weight (g)	D7 weight (g)	Weight change (%)
MeOH	100	21.00 ± 0.41	18.25 ± 0.63	-15.25 ± 1.76^a**^
	250	20.00 ± 9.13	18.00 ± 0.58	-10.99 ± 2.04^a**^
	500	21.00 ± 0.45	19.20 ± 0.66	-9.64 ± 2.32^a**^
AE	100	21.00 ± 0.41	17.00 ± 0.58	-24.11 ± 3.00^a^
	250	20.50 ± 0.65	18.00 ± 0.58	-15.24 ± 1.24^a*^
	500	21.00 ± 0.41	19.75 ± 0.25	-6.32 ± 1.22^a*^
NC	10 ml	21.40 ± 0.68	15.60 ± 0.40	-37.30 ± 3.95
CQ	10 mg	21.20 ± 0.37	22.00 ± 0.63	3.40 ± 2.60^a**^

Data are expressed as means ± SEM; n=5; ^a^ compared to control; ^*^: p<0.01; ^**^: p<0.001; ^***^: p<0.05; D0 to pre-treatment value on day zero; D7 to post-treatment value on day seven; NC: negative control; CQ: chloroquine; MeOH: methanol; AE: aqueous extract

## Discussion

For decades, nature has been the source of therapeutic agents, and based on their traditional use, new drugs have been discovered from natural sources [41]. Due to accelerating rate in drug resistance of common drugs in malaria endemic regions, the necessity to find and create alternative antimalarial medications is unavoidable [46]. The choice of *A. marina* plant was informed by previous reports of their antiplasmodial activities [21,34,37]. The phytochemical screening of the extracts of *A. marina*, a mangrove plant found along coast line of Kenya revealed presence of several bioactive compounds with antiplasmodial activities; flavanoids, tannins, saponin, triterpenoids, glycosides, anthraquinones and alkaloids. These metabolites were also reported in similar studies [47,48]. Similar phytochemicals have also been found in other plant extracts which exhibited antimalarial activity [49,50].

Absence of phenolic and steroid compounds contradicts similar study done in Egypt which reported their presence [48]. Also, the presence of saponin in the present study contradicts a previous study conducted elsewhere which reported absence of saponin [47]. Similarly, anthraquinones and anthocyanine found in the present study were not found in a similar study [37]. The differences in the quantity and composition of secondary metabolites in plants may be influenced by a variety of abiotic environmental factors, including geographic factors (latitude, altitude), sunlight, and climatic, edaphic, and biotic interactions [51]. Additionally, plant developmental stage, post-harvest handling, such as duration of storage, exposure to light, pest and pathogen attack, and storage temperature, are variables that may influence phytochemical variations [52].

Although rodents malaria do not produce exact signs and symptoms as observed in human plasmodial infections, *Plasmodium berghei* infections in mice are reported to cause pathological signs and symptoms akin to human malaria [53]. Some of the available anti-malarial drugs (halofantrine, chloroquine, mefloquine, and artemisinin derivatives) have been identified through use of *in vivo* models [54]. *In-vivo* antiplasmodial activity models were used in this study because they account for prodrug effects as well as the immune system's potential role in infection eradication [55]. Therefore, Swiss albino mice infected with chloroquine sensitive *P. berghei* were used as experimental model to test the antiplasmodial efficacy of stem bark extracts of *A. marina*.

The fact that no mortality was observed at the highest oral dose of 5000 mg/kg body weight of the aqueous and methanolic stem bark extracts of *A. marina* indicated that the mice were safe at the high limit dose as recommended by Organization for Economic Cooperation and Development (OECD) guideline no 425 on acute toxicity [39]. Similar safety findings were reported on similar plant [56,57]. The aqueous and methanolic stem bark extracts of *A. Africana*, a species of the plant that is closely related, produced similar safety results [50]. However, it will be difficult to extrapolate the safety to humans due to species variations [58].

Three models (4-day suppressive, curative and prophylactic tests) are primarily used to test the antiplasmodial effects of the plant [59]. When compared to the negative control group, crude extracts significantly (p<0.05) reduced the early (schizont) stage development of *P. berghei*. The group treated with MeOH500 mg/kg experienced the maximum suppression (93%); this finding suggests that active metabolites increase as dose is increased. According to a prior *in vitro* investigation on the same plant, the extracts suppressed growth at higher concentrations, and our result is consistent with that finding [34]. Given the closeness of suppression levels in aqueous and methanolic extracts at 250 mg/kg and 500 mg/kg, it is possible that the 250 mg/kg dose is the optimal therapeutic dose in mice [60].

Both aqueous and methanolic extracts significantly (p<0.001) reduced parasitemia in the curative test for established infection dose dependently as compared to the negative control. The highest suppression occurred in MeOH 500mg/kg (63.25%). A good prophylactic activity of both aqueous and methanolic *A. marina* extracts was observed in the rodent malaria model. In this study, the 100 mg/kg dose may be the ideal therapeutic dose in mice based on the proximity of suppression in curative and prophylactic testing values at 100 mg/kg, 250 mg/kg, and 500 mg/kg body weight. This is similar to the research conducted by Alli *et al*. [60].

Our study's significant (p<0.05) dose-dependent suppression, curative, and prophylactic effects of *A. marina* are consistent with other studies [50,61]. Neither the conventional medication (CQ 10mg/kg) nor the crude extracts entirely eliminated the parasitemia. This might be due to recrudescence potential of *P. berghei* parasites or short half-lives of some bioactive compounds [62,63]. A previous *in vitro* study elsewhere showed that leaf and bark extracts of *A. marina* has significant antiplasmodial activity [64]. To the best of our knowledge, a similar *in vivo* study on the antiplasmodial efficacies of *A. marina* has not been documented, thus making this the first one.

Potential antiplasmodial agent is considered as moderate, good and very good, if the suppression is ≥ 50% at 500 mg/kg, 250 mg/kg and 100 mg/kg body weight, respectively [42]. Based on this criterion, both aqueous and methanolic extracts from *A. marina* exhibited good activities in curative and prophylactic tests. Moderate activities were observed in the suppressive test. Moderately efficacious herbs are known to lessen the risks of mortality, alleviate malaria symptoms such as fever and pain, and abate the duration of illness by reducing anaemia as well as boosting the host´s immune system thus acting as immunomodulators [65]. Different studies have showed promising antiplasmodial activity of mangrove species *A. germinan, A. officinalis*, and *A. Africana* that are closely related to *A. marina*[26,50,64,66,67].

The antiplasmodial activities of the extracts could be attributed to different effects; individually or in synergy with bioactive compounds such as tannins, alkaloids, terpenoids, steroids, tannins, anthraquinones and flavonoids [43,68]. Alkaloids exhibits antiplasmodial activities by blocking protein synthesis in *P. falciparum* [18]. Quinine, one of the first and most effective antimalarial medications, is an example of an alkaloid and is derived from *cinchona* bark [62]. Flavonoids act by chelating the parasite nucleic acid base pairing [69]. Tannins inactivate microbial adhesins and cell envelope transport proteins [70]. Anthraquinones molecules intercalate into deoxyribonucleic acid (DNA) of microbes, stabilize the covalent intermediate complex of the topo II reaction, causing cell death through apoptosis [71]. Antiplasmodial effects of terpenoids and their derivatives are linked to endoperoxidation of microbes [72]. Likewise, antioxidant properties of bioactive compounds has also been found to be responsible for antiplasmodial activity [41,73]. Antioxidant metabolites impedes the polymerization of heme, which is lethal for the intra-erythrocyte parasite [74]. Indirect immune modulations and other target pathways which are not fully elucidated might also be responsible for parasite suppression [19,75].

Reduction in body weight is a typical feature of malaria in mice, thus a good antimalarial agent should protect plasmodium infected mice against weight loss [54]. When compared to the negative control, the crude extracts failed to significantly (p>0.05) shield mice from weight loss brought on by the malaria parasite in the suppressive test. This might be explained by metabolic processes and hypoglycemia brought on by malaria infection [76]. But when compared to a negative control, extracts from curative and preventative tests significantly (p<0.05) prevented weight loss in the sick mice. The parasite clearance in treated animals, the higher calorie intake, and the enhanced metabolite-related metabolic performance could all have contributed to this [77].

The anti-plasmodial effectiveness of plant extracts is also assessed using the mean survival time [78]. The extracts in suppressive, curative and prophylactic tests significantly (p<0.05) increased the survival time of the experimental mice compared to the negative control group. In another investigation, similar finding was made [72]. This demonstrates the extracts' capacity for protection as the plant repressed *P. berghei* and reduced the parasite's overall pathological consequences in the mice [72].

## Conclusion

The study found that stem bark extracts of *Avicennia marina* significantly reduced *Plasmodium berghei* activity in a mice model. Furthermore, extracts of this plant at tested doses posed no health risk, supporting the use of these compounds in the treatment of rodent malaria. However, studies with human *Plasmodium* strains are suggested.

### What is known about this topic

- Avicennia marina is a mangrove plant specie;
- Studies done in some Asian and African countries have shown that it possesses chemotherapeutic properties.

### What this study adds

- Our finding points to the possible presence of potentially effective antiplasmodial constituents in the stem bark of Kenyan Avicennia marina that suppress parasitemia in vivo models;
- Our findings may serve as the foundation for further investigation of Avicennia marina's antiplasmodial compounds.
